# Health-risk behavior differences between boarding and non-resident students: Brazilian adolescent National School Health Survey

**DOI:** 10.1186/s13690-020-0392-7

**Published:** 2020-03-04

**Authors:** Matias Noll, Priscilla Rayanne e Silva Noll, Carlos Leandro Tiggemann, Daniela Costa Custodio, Erika Aparecida Silveira

**Affiliations:** 1 Instituto Federal Goiano (IF Goiano), Goiás, Brazil; 2 Postgraduate Program in Health Sciences, Faculdade de Medicina, Universidade Federal de Goiás (UFG), Goiás, Brazil; 3 Disciplina de Obstetrícia, Departamento de Obstetrícia e Ginecologia, Faculdade de Medicina, Universidade de São Paulo (FMUSP), Universidade de São Paulo, São Paulo, Brazil; 4 Universidade do Vale do Taquari (UNIVATES), Rio Grande do Sul, Brazil

**Keywords:** Adolescent, Health, Boarding school, Survey, Sexual intercourse, Smoking, Drinking alcohol drinking, Drugs

## Abstract

**Background:**

Studies that evaluated health-risk behaviors with boarding students are scarce. There are no studies with representative samples among adolescents residing in educational institutions in Latin America. To better assess the role of resident status on such behaviors, this study aimed to compare health-risk behaviors between boarding and non-resident students assessed by the Brazilian National Adolescent School Health Survey (PeNSE).

**Methods:**

A cross-sectional study was conducted using the 2015 PeNSE database. A sample of 101,788 students (aged 11–19 years) from both public and private schools throughout all the Brazilian states completed the survey. A self-administered questionnaire was used to evaluate multiple health-related behaviors (sociodemographic characteristics; sexual behavior; cigarette use; drug use; and alcohol use). Poisson regression model-based analyses were performed and the effects measured through the prevalence ratio (PR) and its 95% confidence interval (CI).

**Results:**

Boarding residents reported more health-risk behaviors than non-residents: previous sexual intercourse (PR 1.17, 1.10–1.25), smoking experience (PR 1.12, 1.03–1.21), monthly smoking frequency (PR 1.68, 1.42–1.99), monthly alcohol intake (PR 2.12, 1.79–2.50), inebriation (PR 1.51, 1.35–1.71), drug use experience (PR 1.23, 1.10–1.38), and monthly drug use frequency (PR 1.59, 1.31–1.94).

**Conclusions:**

Boarding residents reported more health-risk behaviors than did non-residents. The results provide insights into an under-researched subject, helping to highlight potential points of intervention for supporting public health programs within the boarding-school student population.

## Background

A high prevalence of several health-risk behaviors is common among adolescent students. This age, between 10 and 19 years [1], is an intense transition phase associated with maturation [2] and several unhealthy behaviors, such as smoking, drinking, illicit drug use, and risky sexual behaviors, begin during this time period [2–4]. Although there is no uniform definition of health-risk behavior worldwide, it is generally considered as behaviors that potentially negatively affect health [5–7], such as substance use, unsafe sexual behavior (e.g., in regard to condom use and number of sexual partners), eating disorders, and antisocial, violent, or suicidal behaviors, among others [8]. Health-risk conditions may be increased by inequalities, which are intensified in socioeconomically weak environments [2, 9–11]. Related to this, students’ living conditions (i.e., school residency environments) can also influence risky health behaviors [12–16].

Health studies with students residing in educational institutions (EI) are scarce [12, 13, 17, 18]. Initial evidence suggests increased alcohol consumption, tobacco and marijuana use, risky sexual behaviors, sedentarism, and poor eating habits among students in residential housing [16, 19–21]. Furthermore, these behaviors tend to increase the longer the student has lived in residential housing [16, 22]. One key explanation could be the feelings of independence from, and lack of accountability toward, their family that students perceive when living away from home [14].

The present study focuses on address the dearth of studies on health-risk behaviors among students residing in EIs within developing Latin American countries, and covered risky sexual behavior and substance use. Only one study with a small, non-representative sample has assessed health behavior risks among adolescents residing in an EI within Latin America [12]. The study revealed that resident Brazilian students reported high levels of unhealthy behaviors, including cigarette smoking, alcohol use, and risky sexual behaviors, which were aggravated by fragile socioeconomic conditions. To better assess the role of resident status on such behaviors, we analyzed data from a national cross-sectional representative sample of students throughout Brazil. Our results could help contribute to planning health promotion and disease prevention activities for addressing problematic health behaviors within this at-risk student sample. Thus, the present study aimed to compare health-risk behaviors between boarding and non-resident students assessed by the Brazilian National School Health Survey (PeNSE).

## Methods

### Overview of research project

This study used data from the 2015 PeNSE [23]. The survey assesses students aged 11–19 classified as 9th graders from both public and private schools throughout Brazil.

The sample size calculations were based on parameters for all 26 states and the Federal District, including inside each state, the capital, and other municipalities with smaller populations. Brazil is conventionally divided into five regions (North, Northeast, Southeast, South, and Midwest) [24]. Samples across capitals and municipalities were random and equiprobabilistic. The following parameters were used for deriving the sample: maximum error of 3%, confidence level of 95%, and prevalence of 0.5. All students in the sample classes were invited to respond to the survey questionnaire. Further information on sampling procedures may be found in Oliveira et al. [24].

### Participants

A total of 120,122 students, attending 4159 classes across 3040 schools, were included in the sample in 2015. From all students enrolled, 15.3% of regular students did not attend the class, did not agree to participate, or did not fully complete the questionnaire. A total of 101,788 students completed the survey questionnaire on the sampling day.

### Ethical considerations

The PeNSE acquired consent from the heads of the schools and all students who voluntarily agreed to participate provided written informed consent. Students were told that they could leave the study at any time and could refuse to participate in any of the procedures. Anonymity and privacy were fully guaranteed to the participants. The PeNSE was approved by the National Commission on Ethics in Research of the National Health Council, which regulates and approves health research involving human participants (no. 1,006,467) [23, 24].

### Data collection and data analysis

Data collection was performed using smartphones distributed to students, who were in class on the day of the interview, by an IBGE technician. The technician explained how to use the device [24].

The explanatory variable evaluated was:

- *Boarding schools*: characterized as regulated educational institutions [25], with students residing at the school during the academic year [18].

The health-risk behavior outcome variables were:

- *Sexual behavior* (prior sexual intercourse; age of first sexual intercourse; number of previous sexual partners; use of condoms during their last instance of sexual intercourse);
- *Substance use:* tobacco use (smoked at least one prior time; frequency of smoking over the past 30 days); alcohol use (experienced drinking at least once; frequency of drinking over the past 30 days, experienced getting drunk at least once); illegal drug use [marijuana, cocaine, crack] (used at least one prior time; frequency of use over the past 30 days);

The adjustment variables included:

- *Sociodemographic characteristics* (municipality [capital city or non-capital city], school [public or private], class shift [full-time or part-time], sex, age, region, mother’s educational history).

Data were analyzed using descriptive statistics and Chi-Square tests (bivariate analysis) to compare frequencies of health-risk behaviors between students residing in EIs (boarding students) and non-residents. Poisson regression models were also analyzed, with the following dummy-coded outcome variables: prior sexual intercourse (0, no; 1, yes), age of first sexual intercourse (0, > 13 years old; 1, ≤ 12 years old), number of sexual partners (0, < 2; 1, ≥ 2); use of condoms during the last instance of sexual intercourse (0, yes; 1, no), experience smoking (0, no; 1, yes), smoked in the last 30 days (0, < 3; 1, ≥ 3 days), experience drinking (0, no; 1, yes), drinking during the last 30 days (0, < 10, 1, ≥ 10 days), experience getting drunk (0, < 3, 1, ≥ 3 days), drug use (0, no; 1, yes), and drug use during the past 30 days (0, < 3, 1, ≥ 3 days).

Poisson regression model-based analysis was performed and the effect measure was the prevalence ratio (PR) with and its 95% confidence interval [26]. Adjusted Poisson regression was performed and the sociodemographic variables were considered confounding variables: municipality, school, class shift, sex, age, region of Brazil, and mother’s schooling (α = .05). Methodological and statistical studies support the inclusion of variables with theoretical grounds in statistical analyses [26–28]. Statistical analyses were performed using SPSS 20.0 (IBM, Armonk, NY, USA).

## Results

Of the 101,788 students who completed the survey, 3.8% (*n* = 3846) resided in an EI and 96.2% (*n* = 97,942) were non-residents. Students attending boarding schools are more prevalent in the north and northeast regions (*p* < .001), non-capital areas (*p* < .001), and public schools (*p* < .001). In addition, boarding students attend more schools with full time classes (*p* < .001) their mothers’ had higher education level categorized more frequently as none or elementary school (*p* < .001) as compared to non-resident students (Table 1).

**Table 1:** Demographic characteristics of the sample from the Brazilian National School Health Survey (PeNSE), Brazil

Variables	Total N (%)	Boarding Students %	Non-residents %	Chi-Square *p*-value
Region (*n* = 101,788)				
North	23,857 (23.4)	27.4	23.3	< .001
Northeast	36,232 (35.6)	34.9	35.6	
Southeast	17,732 (17.4)	14.8	17.5	
Midwest	14,144 (13.9)	15.6	13.8	
South	9823 (9.7)	7.3	9.7	
Municipality (*n* = 101,788)				
Non-capital	50,725 (49.8)	55,5	49,6	< .001
Capital	51,063 (50.2)	44,5	50,4	
School (*n* = 101,788)				
Public	80,905 (79.5)	87.5	79.2	< .001
Private	20,883 (20.5)	12.5	20.8	
Class shift (*n* = 101,378)				
Part-time	78,587 (77.5)	38.9	79	< .001
Full-time (≥ 7 h per day)	22,791 (22.5)	61.1	21	
Mother’s education (*n* = 76,477)				
None	5508 (7.2)	17.5	6.8	< .001
Elementary School^a^	24,181 (31.6)	39.8	31.3	
High School^a^	24,138 (31.6)	24.2	31.8	
Graduate^a^	22,650 (29.6)	18.6	30	
Age (years) (*n* = 101,788)				
11–13	17,205 (16.9)	9.8	17.2	< .001
14	51,480 (50.6)	39.5	51	
15	20,802 (20.4)	27	20.2	
16–19	12,301 (12.1)	23.6	11.6	
Sex (*n* = 101,788)				
Male	49,151 (48.3)	58.7	47.9	< .001
Female	52,637 (51.7)	41.3	52.1	

^a^ Complete and incomplete educational level

Boarding students reported a higher prevalence of sexual intercourse (*p* < .001), more sexual partners (*p* < .001), more smoking experience, and higher monthly smoking frequency (*p* < .001), higher alcohol intake in the past month, as well as drinking to excess (*p* < .001), more drug use experience, and higher monthly drug use (*p* < .001) (Table 2).

**Table 2:** Health-risk behavior comparisons between boarding and non-resident students in Educational Institutions from the Brazilian National School Health Survey (PeNSE), Brazil

Variables ^a^	Total N (%)	Boarding Students %	Non-residents %	Chi-Square *p*-value
Sexual Behavior				
Prior sexual intercourse (*n* = 101,314)				
No	72,815 (71.9)	57.9	72.4	**< .001**
Yes	28,499 (28.1)	42.1	27.6	
Age of first sexual intercourse ^b^ (*n* = 28,271)				
≤ 12 years	7382 (26.1)	29	25.9	**< .001**
13 and 14 years	15,423 (54.6)	45.5	55.1	
≥ 15 years	5466 (19.3)	25.5	19	
Number of sexual partners ^b^ (*n* = 28,327)				
1	10,193 (36)	29.2	36.4	**< .001**
2 to 3	9143 (32.2)	31	32.3	
4 or more	8991 (31.7)	39.7	31.3	
Condom use during last sexual intercourse ^b^ (*n* = 27,335)				
Yes	18,905 (69.2)	69.4	69.1	.850
No	8430 (30.8)	30.6	30.9	
Substance use				
Experience smoking (*n* = 101,626)				
No	82,945 (81.6)	76.3	81.8	**< .001**
Yes	18,681 (18.4)	23.7	18.2	
Smoked during the last 30 days ^b^ (*n* = 18,623)				
None	13,216 (71)	56.6	71.7	**< .001**
1 to 2 days	2878 (15.5)	21.2	15.2	
3 to 9	1322 (7.1)	9.4	7	
10 or more	1207 (6.5)	12.8	6	
Experience drinking (n = 101,591)				
No	48,998 (51.8)	48	48.2	.785
Yes	52,593 (48.2)	52	51.8	
Drinking during the last 30 days ^b^ (*n* = 52,503)				
None	30,355 (57.8)	49.5	58.1	**< .001**
1 to 9 days	19,470 (37.1)	39.3	37	
10 or more	2678 (5.1)	11.3	4.9	
Experience getting drunk ^b^ (*n* = 52,510)				
None	32,292 (61.5)	52.4	61.9	**< .001**
1 to 2 days	13,316 (25.4)	27.1	25.3	
3 to 9 days	4705 (9)	11	8.9	
10 or more	2192 (4.2)	9.5	4	
Previous drug use (marijuana, cocaine, crack) (*n* = 101,544)				
No	92,906 (91.5)	87.9	91.6	**< .001**
Yes	8638 (8.5)	12.1	8.4	
Used drugs in the last 30 days ^b^ (*n* = 8597)				
none	4686 (54.5)	37.9	55.4	**< .001**
1 to 2 days	1990 (23.1)	28.9	22.8	
3 to 9 days	1093 (12.7)	16.6	12.5	
10 or more	828 (9.6)	16.6	9.2	

^a^ For some variables, the totals are less than the total because of missing data; ^b^ Only where applicable

The results of the Poisson regression analyses are presented in Tables 3 and 4. The adjusted PR results indicated that boarding students had more sexual intercourse (PR 1.17, 1.10–1.25), higher experience smoking (PR 1.12, 1.03–1.21), higher monthly smoking frequency (PR 1.68, 1.42–1.99), higher monthly alcohol consumption (PR 2.12, 1.79–2.50), more frequent occasions of getting drunk (PR 1.51, 1.35–1.71), higher previous drug use (PR 1.23, 1.10–1.38), and higher monthly drug use (PR 1.59, 1.31–1.94) as compared to non-resident students.

**Table 3:** Poisson regression results for sexual behaviors; Brazilian National School Health Survey (PeNSE), Brazil

Residing in EIs	Sexual intercourse (yes)			Age of first sexual intercourse (≤12 years old)			Number of sexual partners (≥2)			Condom use during last sexual intercourse (no)		
	Prevalence (%)	PR (95% CI)	PR adj (95% CI)	Prevalence (%)	PR (95% CI)	PR adj (95% CI)	Prevalence (%)	PR (95% CI)	PR adj (95% CI)	Prevalence (%)	PR (95% CI)	PR adj (95% CI)
Non-residents	27.6	1	1	25.9	1	1	63.6	1	1	30.9	1	1
Boarding students	42.1	**1.53(1.45–1.60)**	**1.17(1.10–1.25)**	29	**1.12(1.01–1.23)**	1.06(0.95–1.19)	70.8	**1.11(1.04–1.18)**	1.05(0.98–1.13)	30.6	0.99(0.90–1.09)	0.99(0.88–1.11)the

The analysis was conducted using the Poisson regression model with robust variance. The effect measure is the PR with its respective 95% CI. The model was adjusted for confounding variables: municipality, school, class shift, sex, age, region of Brazil and mother’s schooling

PR adj: adjusted prevalence ratio; CI: confidence interval. EIs: Educational Institutions

**Bolded** *p*-values denote significance (*p* < .05)

**Table 4:** Poisson regression results for substance use; Brazilian National School Health Survey (PeNSE), Brazil

Residing in EIs	Experience smoking (yes)		Smoked during the last 30 days (≥ 3 days)				Experience drinking (yes)		Drinking during the last 30 days (≥ 10 days)			
	Prevalence (%)	PR (95% CI)	PR adj (95% CI)	Prevalence (%)	PR (95% CI)	PR adj (95% CI)	Prevalence (%)	PR (95% CI)	PR adj (95% CI)	Prevalence (%)	PR (95% CI)	PR adj (95% CI)
Non-residents	18.2	1	1	13.1	1	1	51.8	1	1	4.7	1	1
Boarding students	23.7	**1.31(1.22–1.39)**	**1.12(1.03–1.21)**	22.3	**1.69(1.47–1.96)**	**1.68(1.42–1.99)**	52	1.00(0.96–1.05)	0.97(0.93–1.01)	11.3	**2.32(2.02–2.66)**	**2.12(1.79–2.50)**
Residing in EIs	Experience getting drunk (≥ 3 days)				Previous drug use (yes)				Used drugs in the last 30 days (≥ 3 days)			
	Prevalence (%)	PR (95% CI)	PR adj (95% CI)		Prevalence (%)	PR (95% CI)	PR adj (95% CI)		Prevalence (%)	PR (95% CI)	PR adj (95% CI)	
Non-residents	12.5	1	1		8.4	1	1		21.7	1	1	
Boarding students	20.1	**1.60(1.45–1.77)**	**1.51(1.35–1.71)**		12.1	**1.45(1.32–1.59)**	**1.23(1.10–1.38)**		33.2	**1.61(1.46–1.77)**	**1.59(1.31–1.94)**	

The analysis was conducted using the Poisson regression model with robust variance. The effect measure is the PR with its respective 95% CI. The model was adjusted for confounding variables: municipality, school, class shift, sex, age, region of Brazil and mother’s schooling

PR adj: adjusted prevalence ratio; CI: confidence interval. EIs: Educational Institutions

**Bolded** p-values denote significance (p < .05)

## Discussion

Boarding students reported greater frequency of health-risk behaviors as compared to non-residents, regarding risky sexual behavior, smoking, and alcohol and drug consumption. The present results are relevant given the lack of research on health-risk behaviors among adolescent students residing in EIs. Thus, our findings could have implications for targeted health promotion/prevention efforts within this population.

One notable aspect of the present study was the relatively small sample of 9th grade boarding students (less than 4% of the total sample). This is not too surprising given that Brazil has relatively few boarding schools in comparison to other countries such as the United States, Australia, United Kingdom [17, 18], Israel [14], and Turkey [29]. Despite the greater presence of boarding schools in these countries, studies on health-risk behaviors in boarding schools are also relatively sparse worldwide. For instance, in Israel, 10% of students live in boarding schools; yet, very few studies have examined mental health and well-being within this population [14].

Our results revealed that boarding students engaged in more sexual intercourses than did non-residents. Similar findings have been observed in college students in the United States [19]. The boarding school environment provides greater freedom and, consequently, perhaps more permissive sexual attitudes [22]. Interestingly, condom use was comparable between the boarding and non-resident students in the present sample (approximately 69%). Condom use is a key component of sexual health, as condom use is associated with lower incidence of sexually transmitted diseases [2]. Condom use in the context of multiple sexual partners often differs between countries; however, worldwide use is often below 50% among adolescents [2]. According to Spring, Moller, and Coons [3], 34% of sexually active high school students reported not using a condom during their most recent sexual encounter.

Boarding residents were also more likely to smoke than non-residents. In terms of percentages, 23.7% of boarding students reported some smoking experience, with 12.8% smoking on 10 or more days in the past month. This was significantly higher than the 6% of non-residents who smoked 10 or more days, and is in keeping with results from prior studies [9, 30], although 18% of adolescents aged 15 years old in Europe report smoking at least once a week [9]. According to Agmon, Zlotnick, and Finkelstein [14], smoking is cited as a normative behavior within boarding schools in Israel, which could be related to adolescents’ difficulties in avoiding all enticing risky behaviors [31]. However, in Turkey, smoking behavior was lowest and similar between boarding students and non-residents (1.9%; 2.2%) [29].

Alcohol consumption, both in terms of frequency during the last 30 days and getting drunk, was also more prevalent among boarding students compared with non-residents. Specifically, 11.3% of boarding students reported drinking on at least 10 days during the last month, and 20.5% reported getting drunk at least three or more times in their lifetime. This result corroborates prior data from a study that evaluated adolescents in high school [12] and those transitioning from high school to college [22]. Although it is important to highlight that in Brazil, the sale of alcoholic drinks is prohibited to those under 18 years old, the students have substantial access to these products [32, 33], just like elsewhere in the world [34]. Furthermore, in Brazil, drinking alcohol is permitted socially, especially for men, because it is related to male power [32, 33].

The 2012 PeNSE [35] reported that 7.3% of adolescents used illicit drugs at least once in their life, and this number increased to 9% in 2015 [36]. In Israel, approximately 75% of students had never smoked marijuana, which was the only drug evaluated in that study [14]. Drug use has been associated with risky behaviors during the transition from high school to college, but not with type of high school residence [22], even when singled out as a common practice within boarding schools [31]. Illicit drug use is a key public-health problem with deep psychological and social consequences, especially for children and adolescents [2]. Being away from one’s family while at boarding school may lead to drug and alcohol consumption for similar reasons that students engage in risky sexual behaviors [3, 19, 20]. It is important to note that smoking, drinking, and illicit drug use are associated with engaging in risky sexual behaviors [3, 9, 37, 38].

A few study limitations should be noted. First, given the cross-sectional nature of our design, we cannot make any causal inferences from our data; however, it highlights the relevant associations between health-risk behaviors in boarding students. Second, we cannot be certain that adolescents’ self-reports truly reflect their actual behaviors. It should be noted that this kind of bias is inherent in all studies that analyze behavior attitudes. Due to potential demand characteristics when reporting, it is possible that we are underestimating or overestimating the actual behaviors. However, given the relatively large sample, it is likely that biases in reporting did not greatly influence our results.

Overall, higher rates of sexual intercourse, higher prevalence of smoking, and alcohol and drug consumption were reported as more prevalent among boarding students as compared to non-residents. It is possible that the greater independence and freedom experienced at this age is potentially problematic [13, 39]. These data suggest that boarding students warrant greater emphasis on health risk screening and preventive counseling. The implication of the study findings is clear that the significant associations between boarding students and risky health behaviors should be considered in policies and guidelines. In this sense, more structured and healthy environments may be necessary for students who live at this kind of residence [13]. These students need more attention, advice, and health-promotion activities to lead them to responsibility and a conscientious use of their freedom. For instance, trained mentors could be provided to help hold the students more accountable [14]. It is also possible that helping students maintain closer contact with their family could help with health promotion. Finally, schools should develop health promotion activities, such as encouraging students to engage in more adaptive and productive behaviors (i.e., cultural activities and sports) to better assist students’ maturation. Clearer policies and guidelines that can be applied on a daily basis will help create a safe and supportive environment for this student population [18, 25].

## Conclusions

Boarding residents reported more health-risk behaviors than did non-residents. Our results provide insights into an under-researched subject, helping to highlight potential points of intervention for supporting public health programs within the boarding-school student population.

## Data Availability

The datasets used and/or analysed during the current study are available from the corresponding author on request.

## Abbreviation

PeNSE: Brazilian National School-Based Health Survey
